# Evaluating the Effect of Ionic Strength on Duplex Stability for PNA Having Negatively or Positively Charged Side Chains

**DOI:** 10.1371/journal.pone.0058670

**Published:** 2013-03-06

**Authors:** N. Tilani S. De Costa, Jennifer M. Heemstra

**Affiliations:** Department of Chemistry and the Center for Cell and Genome Science, University of Utah, Salt Lake City, Utah, United States of America; University of Quebect at Trois-Rivieres, Canada

## Abstract

The enhanced thermodynamic stability of PNA:DNA and PNA:RNA duplexes compared with DNA:DNA and DNA:RNA duplexes has been attributed in part to the lack of electrostatic repulsion between the uncharged PNA backbone and negatively charged DNA or RNA backbone. However, there are no previously reported studies that systematically evaluate the effect of ionic strength on duplex stability for PNA having a charged backbone. Here we investigate the role of charge repulsion in PNA binding by synthesizing PNA strands having negatively or positively charged side chains, then measuring their duplex stability with DNA or RNA at varying salt concentrations. At low salt concentrations, positively charged PNA binds more strongly to DNA and RNA than does negatively charged PNA. However, at medium to high salt concentrations, this trend is reversed, and negatively charged PNA shows higher affinity for DNA and RNA than does positively charged PNA. These results show that charge screening by counterions in solution enables negatively charged side chains to be incorporated into the PNA backbone without reducing duplex stability with DNA and RNA. This research provides new insight into the role of electrostatics in PNA binding, and demonstrates that introduction of negatively charged side chains is not significantly detrimental to PNA binding affinity at physiological ionic strength. The ability to incorporate negative charge without sacrificing binding affinity is anticipated to enable the development of PNA therapeutics that take advantage of both the inherent benefits of PNA and the multitude of charge-based delivery technologies currently being developed for DNA and RNA.

## Introduction

**Figure 1:**
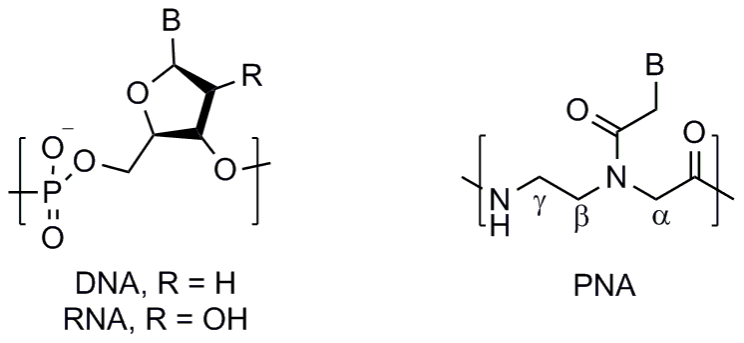
Chemical structures of DNA, RNA and PNA.

Peptide nucleic acid (PNA) [1] is an artificial nucleic acid having unique physicochemical properties, which can largely be attributed to the fact that PNA has an achiral, peptide-like *N*-(2-aminoethyl)glycine backbone in place of the sugar-phosphate backbone found in DNA and RNA (Figure 1). PNA shows tremendous potential for use in molecular diagnostics and antisense therapeutics [2]–[4] due to its greater binding affinity, selectivity, [5] and strand-invasion capability [6]–[11] relative to native nucleic acids, as well as its resistance to degradation by nucleases and proteases. [12] The enhanced thermodynamic stability of PNA:DNA and PNA:RNA duplexes compared with DNA:DNA and DNA:RNA duplexes has been attributed in part to the lack of electrostatic repulsion between the uncharged PNA backbone and negatively charged DNA or RNA backbone. [5] However, there are no reported studies that systematically evaluate the effect of ionic strength on duplex stability for PNA having a charged backbone.

**Figure 2:**
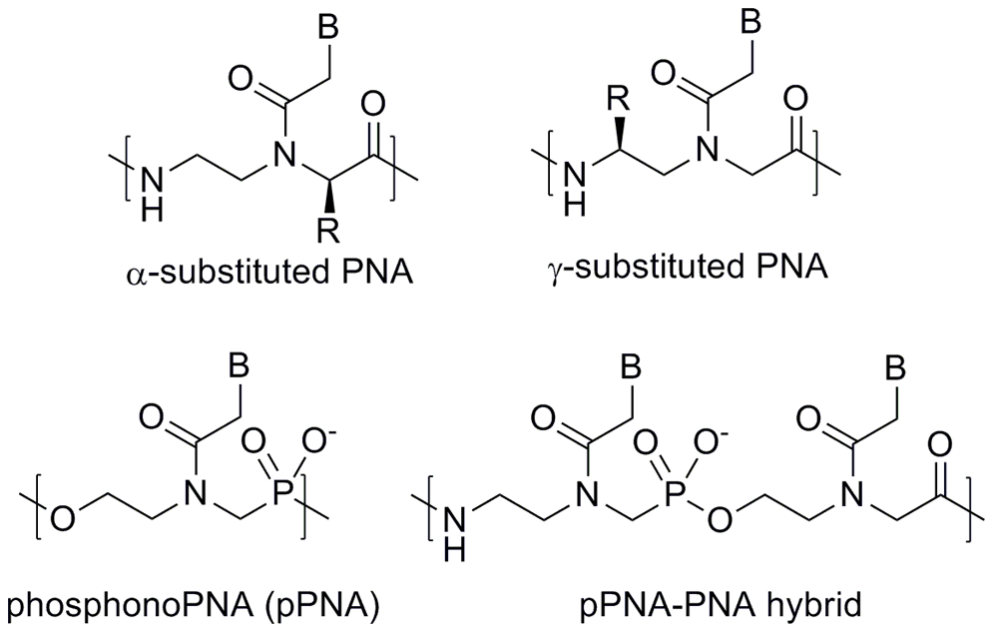
Chemical structures of backbone-modified PNA.

Previous studies have shown that incorporation of negatively charged or neutral side chains at the α-position (Figure 2) of the PNA backbone reduces binding affinity with DNA, whereas incorporation of positively charged side chains increases binding affinity with DNA and has negligible effect on binding affinity with RNA. [13]–[15] However, these studies were only carried out at a single salt concentration, and binding affinity of negatively charged PNA with RNA was not studied. In the case of γ-substituted PNA, positively charged or neutral side chains increase binding affinity with DNA, [16]–[20] but this increase is primarily attributed to steric or hydrogen-bonding effects leading to conformational preorganization of the PNA backbone. [21], [22] There is evidence that negatively charged side chains are also tolerated at the γ-position, [17], [23] but their effect on binding affinity with DNA at varying ionic strength has not been thoroughly studied. Additionally, the binding properties of γ-substituted PNA with RNA have only been minimally investigated. [16]

Taking a different approach to charge incorporation, the research groups of Peyman and Efimov independently synthesized and studied phosphonoPNA (pPNA/PHONA), having a negatively charged phosphate group inserted into the PNA backbone (Figure 2). [24]–[26] pPNA:DNA and pPNA:RNA duplexes were found to have T_m_ values significantly lower than those of PNA:DNA and PNA:RNA, and in fact even lower than those of the corresponding DNA:DNA and DNA:RNA duplexes. However, alternating pPNA monomers with PNA monomers to give a pPNA-PNA hybrid resulted in duplex stabilities with DNA and RNA that approached those of PNA:DNA and PNA:RNA. [26] The results of these studies could be interpreted to conclude that increasing negative charge decreases PNA duplex stability via electrostatic repulsion. However, it is important to note that the backbone conformation of pPNA is likely to differ significantly from that of PNA and γ-substituted PNA. Thus, the decreased duplex stability of pPNA may result predominantly from structural, rather than electrostatic effects.

Here we present the first detailed investigation of the effect of ionic strength on binding affinity of charged PNA, and show that charge screening of electrostatic repulsion by counterions in solution enables negatively charged side chains to be incorporated into the PNA backbone without reducing duplex stability with DNA and RNA. Thus, electrostatic interactions do play a role in PNA binding, but this effect is manifested in differential salt dependence, such that at medium to high salt concentrations, negatively charged PNA actually binds more strongly to DNA and RNA than does positively charged PNA.

## Materials and Methods

### Monomer synthesis

**Figure 3:**
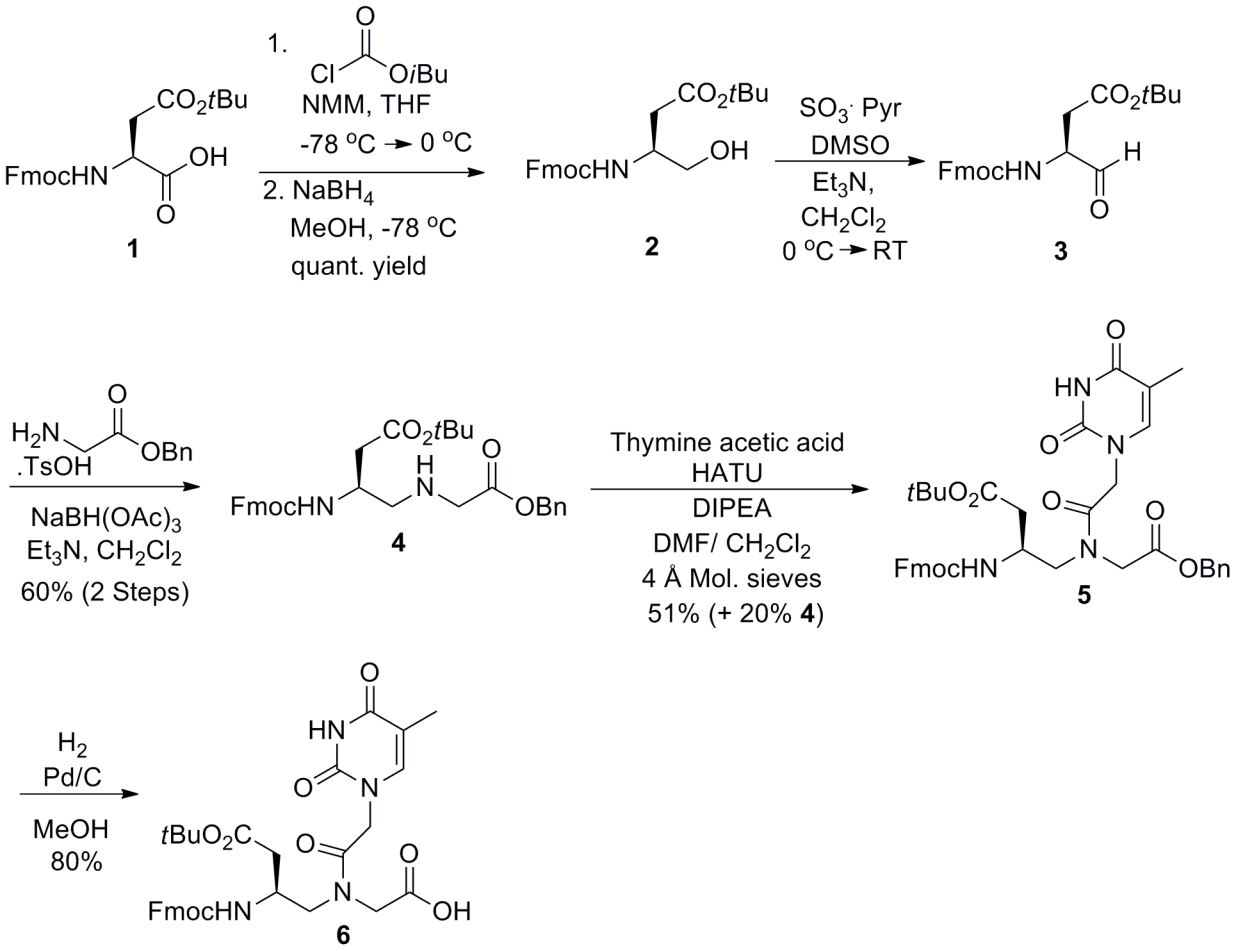
Synthesis of negatively charged PNA monomer.

Positively charged PNA monomer and γ-methyl substituted PNA monomer were synthesized from Fmoc-L-Lys(Boc)-OH and Fmoc-L-Ala-OH respectively using previously reported procedures. [17], [27] A similar procedure was employed for synthesis of the negatively charged PNA monomer (Figure 3). Commercially available Fmoc-L-aspartic acid β-*tert*-butyl ester **1** was reduced to give the corresponding alcohol **2** [28] in quantitative yield, which was subsequently subjected to Parikh-Doering conditions [29] to give the aldehyde **3**. [30] Aldehyde **3** was immediately subjected to the reductive amination with glycine benzyl ester 4-toluenesulfonate to afford negatively charged PNA backbone **4**. Subsequent coupling of **4** with thymine-1-acetic acid under HATU/DIPEA afforded the amide **5**. Final removal of the benzyl ester via hydrogenation afforded negatively charged PNA monomer **6**.

### Oligomer synthesis

PNA oligomers were synthesized on NovaSyn TGR R resin (0.2 mmol/g) according to published procedures using manual or semi-automated (Activo P-14 Peptide Synthesizer) solid-phase peptide synthesis. [31]–[35] The oligomers were cleaved from the resin using TFA:triisopropylsilane:H_2_O (95:2.5:2.5). The resulting mixtures were precipitated with ether, purified by RP-HPLC (Agilent ZORBAX 300SB-C18, 5 µM particle size, 9.4×250 mm) with a binary mixture of 0.1% TFA in water (eluent A) and 0.1% TFA in CH_3_CN (eluent B). The linear gradient was 8–18% of eluent B for 26 min at 50°C at a flow rate of 4.0 mL/min. A small fraction of the purified compound was reinjected to RP-HPLC (Agilent ZORBAX 300SB-C18, 5 µM particle size, 4.6×250 mm) for analysis. The linear gradient was 8–18% of eluent B for 26 min at 50°C at a flow rate of 1.0 mL/min. PNA strands were characterized by MALDI-TOF mass spectrometry in reflectron positive mode using a Waters Micromass MALDI Micro MX (see Figure S2, Figure S3, Figure S4, Figure S5, Figure S6, Figure S7). The concentrations of the PNA oligomers were determined from the OD at 260 nm recorded in a UV-VIS Spectrophotometer (SHIMADZU 1800), using the extinction coefficient 100,300 M^−1^cm^−1^ for the sequence GTAGATCACT.

### Buffer preparation

Varying concentrations of NaCl were added to 10 mM phosphate buffer, pH 7.6, and initial pH measured. pH was adjusted to 7.2 using 6 M HCl or 5 M NaOH. The change of Na^+^ concentration in the buffer due to NaOH is equal to or less than 0.4%.

### UV-melting studies

All samples were prepared in buffer containing 10 mM sodium phosphate, pH 7.2, with added NaCl (0, 50, 100, 250, 500 1000 mM), except for the physiological buffer, which was 0.5 mM MgCl_2_, 137 mM NaCl, 2.7 mM KCl, 1.5 mM KH_2_PO_4_, 8.1 mM Na_2_HPO_4_, pH 7.4. The samples were incubated at 95°C for 1 min, followed by gradual cooling to room temperature using a BioRad-MJ Mini Personal Thermal Cycler, before data collection. UV-Vis absorbance at 260 nm was recorded and corrected using the absorbance at 380 nm (UV cell path length  =  1 cm). The data were recorded at a rate of 1°C/min, in 0.5°C intervals, for both the heating (20–80°C) and cooling (80–20°C) runs, (except PNA **3pos** data was recorded for heating (20–90°C) and reverse). The T_m_ values were determined by taking the first derivative of the cooling profiles, using Origin 8.5.1 software. Final T_m_ is an average of three or four independent trials, and error bars represent the standard deviation.

### Thermodynamic analysis

The UV melting data were analyzed to obtain van't Hoff transition enthalpies.[36], [37] Baseline correction was applied to each plot of normalized absorbance vs temperature, providing plots of fraction melted (θ) vs temperature. The thermodynamic parameters were determined by plotting ln *K_a_* vs 1/T (van't Hoff plot). Values of *K_a_*, the affinity constant, at each temperature were determined using the following equation for bimolecular, complementary oligonucleotides, where *C*_o_ is the initial strand concentration.



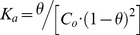
For a two-state transition, if Δ*H* is independent of the temperature, then a plot of ln *K_a_* vs 1/T is linear, giving -Δ*H*/R as the slope and Δ*S*/R as the y-intercept. Gibbs free energy (Δ*G*) was calculated using the following equation, where T =  298 K.



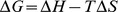



## Results

### Structure and sequence of positively and negatively charged PNA strands

**Figure 4:**
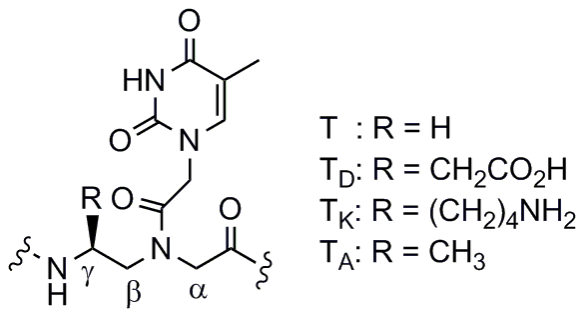
Chemical structure of γ-substituted PNA monomers.

To investigate the effect of ionic strength on duplex stability for charged PNA, negatively and positively charged PNA monomers were synthesized using L-Asp [38] and L-Lys [17] residues, respectively, to construct the ethylenediamine portion of the PNA backbone (Figure 4). Substitution at the γ-position is known to be advantageous over substitution at the α-position, with regard to binding affinity, unambiguous antiparallel binding, and helical induction. [16]–[22] Specifically, an (*S*)-stereocenter at the γ-position conformationally preorganizes the PNA backbone into a right-handed helix, which is favorable for binding to DNA and RNA. This stereoinduction is unidirectional from C- to N-terminus, resulting in antiparallel sequence alignment, and projects the γ-substituents away from the backbone. We used the Nielsen decamer sequence H-GTAGATCACT-NH_2_ [5] for the current study, as its hybridization to DNA and RNA has been thoroughly investigated. Additionally, this sequence contains three equally-spaced thymine residues as convenient points for substitution with our charged monomers. Solid-phase peptide synthesis [35] was used to generate nonfunctionalized PNA (PNA **nf**), as well as PNA strands containing either one or three positively charged (PNA **1pos**/**3pos**) or negatively charged (PNA **1neg**/**3neg**) monomers (Table 1). With these sequences in hand, we investigated their thermal melting behaviour with complementary DNA (DNA **1**) and RNA (RNA **1**) at varying salt concentrations.

**Table 1:** PNA, DNA and RNA Sequences.

Name	Sequence
PNA nf	H-GTAGATCACT-NH_2_
PNA 1neg	H-GTAGAT_D_CACT-NH_2_
PNA 3neg	H-GT_D_AGAT_D_CACT_D_-NH_2_
PNA 1pos	H-GTAGAT_K_CACT-NH_2_
PNA 3pos	H-GT_K_AGAT_K_CACT_K_-NH_2_
PNA 1Me DNA 1	H-GTAGAT_A_CACT-NH_2_ 5′-AGTGATCTAC-3′
DNA 2	5′-GTAGATCACT-3′
RNA 1	5′-AGUGAUCUAC-3′

### The effect of ionic strength on duplex stability for DNA, RNA and PNA

**Figure 5:**
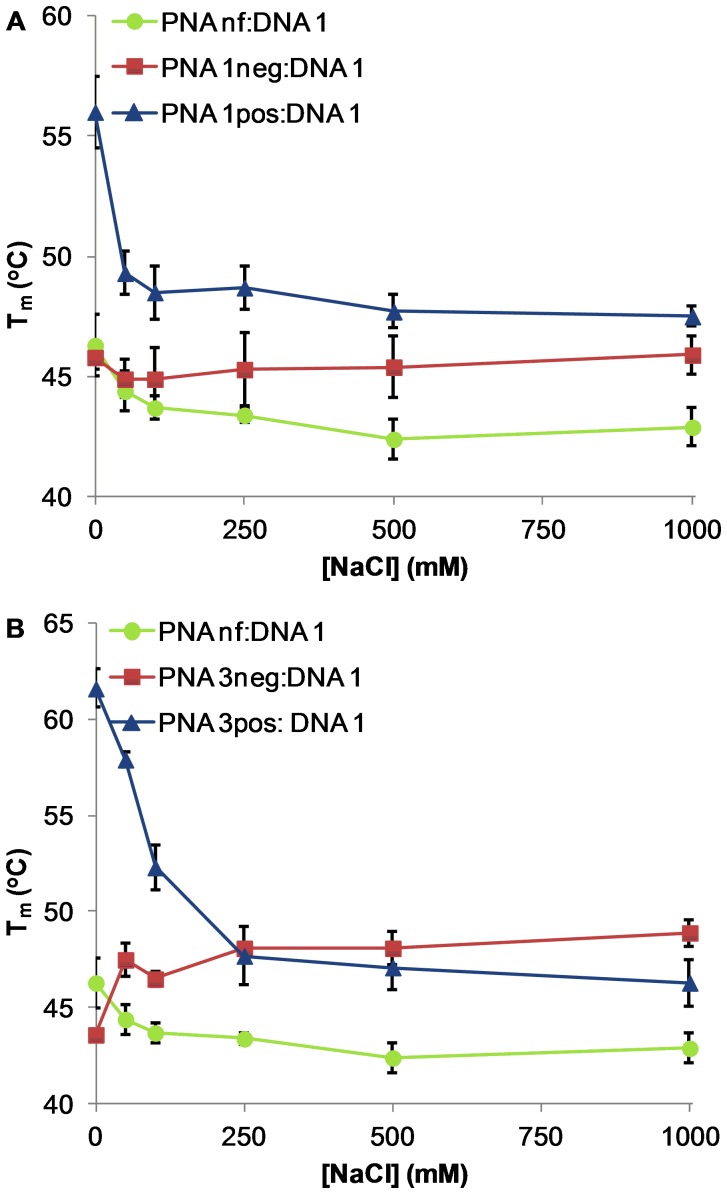
T_m_ vs [NaCl] for PNA:DNA 1 duplexes. (A) PNA (**nf**/**1neg**/**1pos**):DNA **1**. (B) PNA (**nf**/**3neg**/**3pos**):DNA **1**. Conditions: 3 µM PNA, 3 µM DNA, 10 mM sodium phosphate buffer with added NaCl, pH 7.2. Error bars represent standard deviation of three or four independent trials.

**Figure 6:**
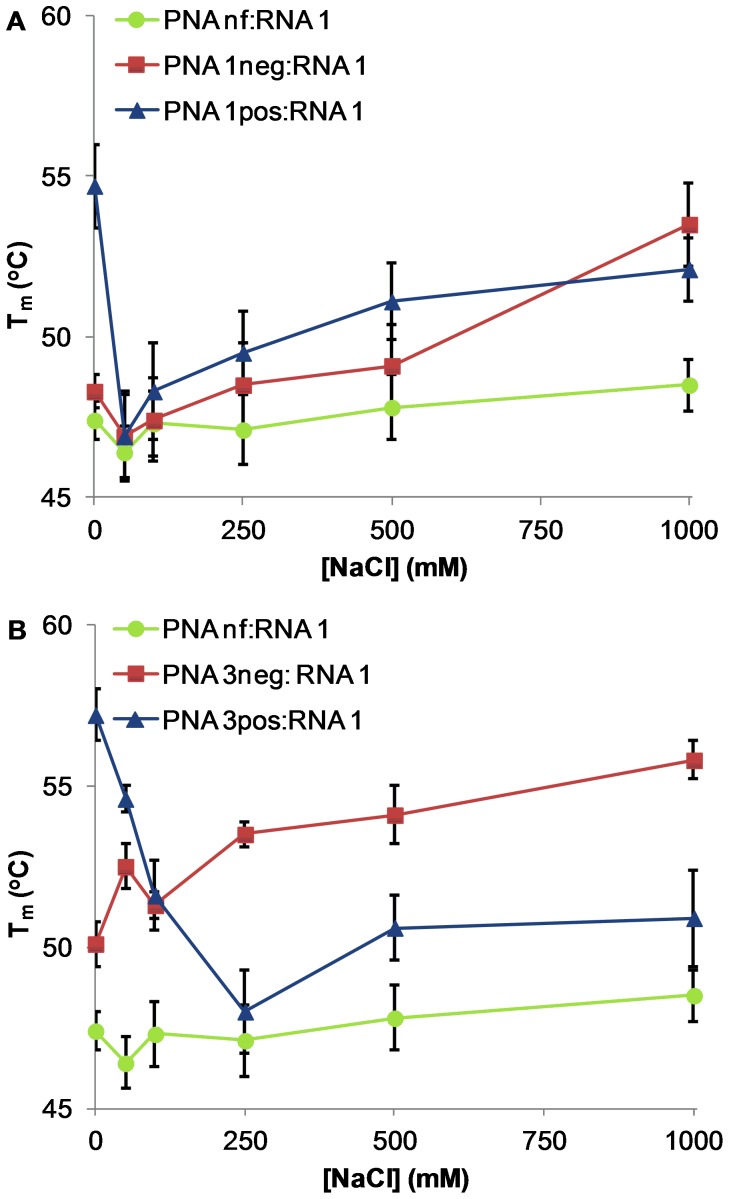
T_m_ vs [NaCl] for PNA:RNA 1 duplexes. (A) PNA (**nf**/**1neg**/**1pos**):RNA **1**. (B) PNA (**nf**/**3neg**/**3pos**):RNA **1**. Conditions: 3 µM PNA, 3 µM RNA, 10 mM sodium phosphate buffer with added NaCl, pH 7.2. Error bars represent standard deviation of three or four independent trials.

DNA:DNA and DNA:RNA duplexes are known to demonstrate positive salt dependence, in which increased ionic strength of the buffer solution leads to increased melting temperature (T_m_) due to charge screening of the electrostatic repulsion between the negatively charged strands. [39] Thus, we were unsurprised to see the T_m_ values of DNA 1:DNA 2 and RNA 1:DNA 2 increase with increasing concentrations of NaCl (Figure S1). In contrast, PNA:DNA duplexes demonstrate negative salt dependence, in which increased ionic strength leads to a decrease in T_m_. [5] The thermodynamic stability of PNA:DNA duplexes has been attributed in part to entropically favorable counterion release upon duplex formation. [40] Therefore, increasing the salt concentration destabilizes the PNA:DNA duplex. However, the efflux of cations in PNA:DNA duplex formation is less than the influx of cations in DNA:DNA duplex formation, so the net salt effect is smaller for PNA:DNA relative to DNA:DNA. As anticipated, the T_m_ of PNA nf:DNA 1 shows a weak negative salt dependence (Figure 5, green line). In the case of PNA:RNA duplexes, ionic strength appears to have little effect on hybridization, as PNA nf:RNA 1 shows neutral salt dependence (Figure 6, green line). In previous work by Ly and coworkers, positively charged guanidinium-PNA (GPNA):DNA duplexes demonstrated negative salt dependence. [18] Also, Romanelli and coworkers have shown that in the case of PNA_2_:DNA triplexes containing negatively charged PNA, doubling salt concentration increases stability. [23] Thus, we anticipated that our negatively charged PNA would demonstrate positive salt dependence in duplex formation with DNA and RNA.

### Duplex stability of charged PNA with DNA at varying salt concentrations

The introduction of a single positive or negative γ-substituent was found to enhance PNA:DNA duplex stability, as PNA 1pos:DNA 1 and PNA 1neg:DNA 1 displayed higher T_m_ values than PNA nf:DNA 1 (Figure 5A). This increase in duplex stability can be attributed primarily to backbone preorganization induced by the γ-substituent, as an analogous PNA strand having a single γ-methyl substituent (PNA 1Me) [9]–[11] demonstrated nearly identical T_m_ values to PNA 1pos (Table 2). Similar to GPNA, PNA 1pos:DNA 1 showed a negative salt dependence with increasing concentrations of NaCl. In contrast, PNA 1neg:DNA 1 showed a neutral salt dependence, providing preliminary evidence that the presence of negative charge in the PNA backbone can result in reversal of salt dependence for duplex formation.

**Table 2:** T_m_ of PNA:DNA **1** duplexes at varying salt concentrations.*

[NaCl] =	0 M	50 mM	100 mM	250 mM	500 mM	1 M
PNA **nf**:DNA **1**	46.3±1.3	44.4±0.8	43.7±0.5	43.4±0.3	42.4±0.8	42.9±0.8
PNA **1neg**:DNA **1**	45.8±0.5	44.9±0.8	44.9±1.3	45.3±1.5	45.4±1.3	45.9±0.8
PNA **3neg**:DNA **1**	43.6±0.1	47.5±0.9	46.5±0.4	48.1±0.3	48.1±0.9	48.9±0.7
PNA **1pos**:DNA **1**	56.0±1.5	49.3±0.9	48.5±1.1	48.7±0.9	47.7±0.7	47.5±0.4
PNA **3pos**:DNA **1**	61.6±1.0	57.9±0.4	52.3±1.2	47.7±1.5	47.1±1.2	46.3±1.2
PNA **1Me**:DNA **1**	52.2±0.8	50.5±0.8	49.8±0.5	49.3±0.5	48.8±0.5	49.0±0.3

* Conditions: 3 µM PNA, 3 µM DNA, 10 mM sodium phosphate buffer with added NaCl, pH 7.2. Errors represent standard deviation of three or four independent trials.

Upon increasing the number of charged residues from one to three, a more pronounced effect on T_m_ was observed (Figure 5B). As anticipated, the duplex stabilities of PNA **3neg** and PNA **3pos** with DNA **1** were greater than that of PNA **nf** with DNA **1**, likely due to backbone preorganization by the γ-substituents. Similar to PNA **1pos**:DNA **1**, PNA **3pos**:DNA **1** showed a negative salt dependence with increasing NaCl concentration. However, as we anticipated, incorporation of three negative charges resulted in a positive salt dependence for PNA **3neg**:DNA **1**, as this duplex is presumably able to take advantage of charge screening when cations are present. Interestingly, in the presence of only 10 mM sodium (from the sodium phosphate buffer), T_m_ values follow the order of PNA **3pos**>PNA **nf**>PNA **3neg**, revealing the effect of unscreened electrostatic contributions. But, with added NaCl concentrations of 250 mM and above, PNA **3neg**:DNA **1** surprisingly becomes more stable than PNA **3pos**:DNA **1** (Table 2).

### Duplex stability of charged PNA with RNA at varying salt concentrations

We next investigated the binding of charged PNA strands with complementary RNA. As was the case for DNA, incorporation of one or three γ-substituted monomers in the PNA sequence increases the overall duplex stability with RNA (Figure 6). Both PNA 1neg:RNA 1 and PNA 1pos:RNA 1 showed an initial decrease in T_m_ going from 0 to 50 mM NaCl, followed by a gradual increase in T_m_ up to 1 M NaCl (Figure 6A). However, the T_m_ values of these two duplexes are the same within error at NaCl concentrations of 50 mM and above, indicating that the presence of a single charged residue has only minimal impact on PNA:RNA binding. Increasing the number of charged residues on PNA from one to three produced a more dramatic effect on RNA binding (Figure 6B). Analogous to the results described above for binding of triply charged PNA with DNA, PNA 3pos:RNA 1 displays a negative salt dependence and PNA 3neg:RNA 1 displays a positive salt dependence. However, in the case of RNA, the threshold for negatively charged PNA to surpass positively charged PNA in binding affinity is much lower at approximately 100 mM NaCl (Table 3).

**Table 3:** T_m_ of PNA:RNA **1** duplexes at varying salt concentrations.*

[NaCl] =	0 M	50 mM	100 mM	250 mM	500 mM	1 M
PNA **nf**:RNA **1**	47.4±0.6	46.4±0.8	47.3±1.0	47.1±1.1	47.8±1.0	48.5±0.8
PNA **1neg**:RNA **1**	48.3±0.5	46.9±1.3	47.4±1.3	48.5±1.3	49.1±1.3	53.5±1.3
PNA **3neg**:RNA **1**	50.1±0.7	52.5±0.7	51.3±0.4	53.5±0.4	54.1±0.9	55.8±0.6
PNA **1pos**:RNA **1**	54.7±1.3	46.9±1.4	48.3±1.5	49.5±1.3	51.1±1.2	52.1±1.0
PNA **3pos**:RNA **1**	57.2±0.8	54.6±0.4	51.6±1.1	48.0±1.3	50.6±1.0	50.9±1.5
PNA **1Me**:RNA **1**	50.5±1.3	50.1±1.1	50.6±1.1	51.6±1.1	53.3±0.9	54.0±1.0

* Conditions: 3 µM PNA, 3 µM RNA, 10 mM sodium phosphate buffer with added NaCl, pH 7.2. Errors represent standard deviation of three or four independent trials.

### Duplex stability of charged PNA with DNA and RNA under physiological salt conditions

Given the increasing use of PNA for *in vivo* applications, we sought to investigate the duplex stability of our charged PNA with DNA and RNA in a buffer that mimics physiological salt conditions (0.5 mM MgCl_2_, 137 mM NaCl, 2.7 mM KCl, 1.5 mM KH_2_PO_4_, 8.1 mM Na_2_HPO_4_, pH 7.4) [41] (Table 4). Consistent with previous observations, negatively charged PNA binds slightly weaker with DNA than does positively charged PNA. However, in the case of RNA binding, the negatively charged PNA was again superior to positively charged PNA when three charged substituents were present on the PNA backbone. These results reinforce the observations outlined above, and lead to the unexpected conclusion that adding negative charge to PNA may in fact increase binding affinity in RNA-targeted antisense therapeutics.

**Table 4:** Tm of PNA:DNA **1** and PNA:RNA **1** duplexes under simulated physiological buffer conditions.*

Complement	T_m_ with DNA 1 (°C)	T_m_ with RNA 1 (°C)
DNA **2**	37.2±0.1	32.5±0.8
PNA **nf**	43.2±0.5	47.1±1.1
PNA **1neg**	45.9±0.8	48.1±1.1
PNA **1pos**	46.9±0.3	46.9±0.8
PNA **3neg**	46.1±0.6	49.9±1.5
PNA **3pos**	49.1±1.0	46.5±1.7

* Conditions: 3 µM PNA, 3 µM DNA or RNA, 0.5 mM MgCl_2_, 137 mM NaCl, 2.7 mM KCl, 1.5 mM KH_2_PO_4_, 8.1 mM Na_2_HPO_4_, pH 7.4. Errors represent standard deviation of three independent trials.

Van't Hoff analysis was performed on the UV melting data to obtain the thermodynamic parameters for duplex formation of PNA **3neg** and PNA **3pos** with DNA and RNA in physiological buffer (Table 5). [36], [37] Unsurprisingly, the Gibbs free energy change (Δ*G*) follows a similar trend as the T_m_ values for the duplexes, with higher free energy gain observed for duplexes having higher values of T_m_. In duplex formation with DNA, PNA **3neg** shows lower enthalpic driving force, but also lower entropic cost, relative to PNA **3pos**. However, in the case of RNA duplex formation, the opposite is true; PNA **3neg** shows higher enthalpic driving force, but higher entropic cost, relative to PNA **3pos**.

**Table 5:** Thermodynamic parameters for the formation of duplexes of PNA 3neg and PNA 3pos with DNA and RNA.*

Duplex	T_m_ (°C)	−Δ*G*(kJ⋅mol^−1^)	−Δ*H*(kJ⋅mol^−1^)	−TΔ*S*(kJ⋅mol^−1^)
PNA **3neg**:DNA **1**	46.1±0.6	48.0±0.3	208.4±1.0	160.4±1.0
PNA **3pos**:DNA **1**	49.1±1.0	49.8±0.3	213.2±2.7	163.4±2.9
PNA **3neg**:RNA **1**	49.9±1.5	49.1±0.8	203.4±3.6	154.3±2.9
PNA **3pos**:RNA **1**	46.5±1.7	47.9±0.3	191.5±0.4	143.6±0.6

* Averages from van't Hoff analysis of three trials of UV melting data. Errors represent standard deviation of three independent trials.

## Discussion

PNA:RNA duplexes adopt the A-form structure preferred by RNA, [42] whereas PNA:DNA duplexes adopt an intermediate structure between A- and B-form. [22], [43], [44] Consequently, PNA:RNA duplexes generally show a higher thermal stability relative to analogous PNA:DNA duplexes. A-form duplexes have been shown to engage in tighter and more structured counterion binding relative to B-form duplexes. [45] Thus, we hypothesize that the structural variation between PNA:DNA and PNA:RNA duplexes is responsible for the increased contribution of PNA backbone charge and NaCl concentration in the case of PNA:RNA binding. This hypothesis is supported by the thermodynamic data in Table 5, where the PNA **3neg**:RNA duplex has greater enthalpic gain, but greater entropic cost, relative to the PNA **3pos**:RNA duplex, as would be anticipated in the case of tight counterion binding to the PNA **3neg**:RNA duplex. We are intrigued by the fact that the charged PNA:RNA duplexes do not follow a logarithmic trend for T_m_ as a function of ionic strength, as is the case for DNA:DNA and DNA:RNA duplexes. [46] Future studies will utilize molecular dynamics simulations to provide greater insight into the effect of PNA charge on duplex structure. Additionally, work is currently underway in our lab to explore the effect of PNA charge density and charge spacing on salt-dependent binding affinity with DNA and RNA.

It should be noted that the Asp and Lys residues used for this initial study have a slight variation in side chain length. However, given the fact that the PNA:DNA helix diameter is approximately 23 Å, [22] and previous studies have reported that the Lys side chains are not involved in non specific charge-charge interactions, [16] the two carbon difference in side chain length is anticipated to have little to no impact on duplex stability. Thus, we attribute the changes in duplex stability for negatively and positively charged PNA primarily to the differential electrostatic properties of these PNA strands.

Given the hypothesis that lack of electrostatic repulsion plays a key role in PNA binding, it is surprising to discover that adding negatively charged side chains to PNA does not significantly decrease binding affinity with DNA and RNA at physiological ionic strength. Moreover, because positively charged PNA displays negative salt dependence and negatively charged PNA displays positive salt dependence, at medium to high salt concentrations, negatively charged PNA actually binds more strongly to DNA and RNA than does positively charged PNA. Presumably, preorganization of the PNA backbone via hydrogen bonding is primarily responsible for the enhanced duplex stability of PNA with DNA and RNA. This hypothesis has been previously reported in the literature, [47], [48] and recent studies by Ganesh and coworkers [20] have demonstrated that additional backbone hydrogen bonding interactions can be used to further increase binding affinity or favor parallel versus antiparallel alignment of the nucleic acid strands.

The recent popularity of antisense therapeutics such as siRNA has prompted the development of a multitude of technologies aimed at enhancing the circulation lifetime and cell permeability of nucleic acids *in vivo*. [49], [50] However, nearly all of these technologies function on the basis of the negatively charged backbone found in native nucleic acids. Thus, the ability to impart negative charge to PNA without sacrificing binding affinity with DNA and RNA may enable the development of therapeutics that are able to take advantage of the delivery technologies described above as well as the inherent benefits of PNA such as increased stability and enhanced binding affinity. [51] This would open the door to previously unexplored nucleic acid-delivery vector combinations, and may lead to the discovery of antisense therapeutics with enhanced *in vivo* efficacy. Studies investigating cellular delivery of negatively charged PNA using charge-based delivery methods are currently underway.

## Supporting Information

Figure S1 **T_m_ vs [NaCl] for DNA 1:DNA 2 and RNA 1:DNA 2 duplexes.** Conditions: 3 µM DNA, 3 µM RNA, 10 mM phosphate buffer with added NaCl, pH 7.2. Error bars represent standard deviation of three independent trials.
(TIF)

Figure S2 HPLC and MALDI-TOF MS of PNA nf (H-GTAGATCACT-NH_2_). *m/z* 2727.48 (calcd [M]^+^ 2727.04).
(TIF)

Figure S3 HPLC and MALDI-TOF MS of PNA 1neg (H-GTAGAT_D_CACT-NH_2_). *m/z* 2785.54 (calcd [M]^+^ 2785.04).
(TIF)

Figure S4 **HPLC and MALDI-TOF MS of PNA 3neg (H-GT_D_AGAT_D_CACT_D_-NH_2_).** *m/z* 2902.67 (calcd [M+H]^+^ 2902.14); 2924.68 (calcd [M+Na]^+^ 2924.12).
(TIF)

Figure S5 **HPLC and MALDI-TOF MS of PNA 1pos (H-GTAGAT_K_CACT-NH_2_).** *m/z* 2800.76 (calcd [M+H]^+^ 2799.12); 2822.71 (calcd [M+Na]^+^ 2821.1).
(TIF)

Figure S6 **HPLC and MALDI-TOF MS of PNA 3pos (H-GT_K_AGAT_K_CACT_K_-NH_2_).** *m/z* 2941.38 (calcd [M+H]^+^ 2941.26); 2963.37 (calcd [M+Na]^+^ 2963.24).
(TIF)

Figure S7 **HPLC and MALDI-TOF MS of PNA 1Me (H-GTAGAT_A_CACT-NH_2_).** *m/z* 2740.99 (calcd [M]^+^ 2741.05); *m/z* 2741.96 (calcd [M+H]^+^ 2942.06); 2763.93 (calcd [M+Na]^+^ 2764.04).
(TIF)

File S1 **General techniques and synthesis of PNA monomers.**
(DOC)
