# Supplementary figures and images for: Evaluating the Effect of Ionic Strength on Duplex Stability for PNA Having Negatively or Positively Charged Side Chains
Source: PLoS One. 2013 Mar 6;8(3):e58670. doi: 10.1371/journal.pone.0058670 (PMC3590165; doi:10.1371/journal.pone.0058670)

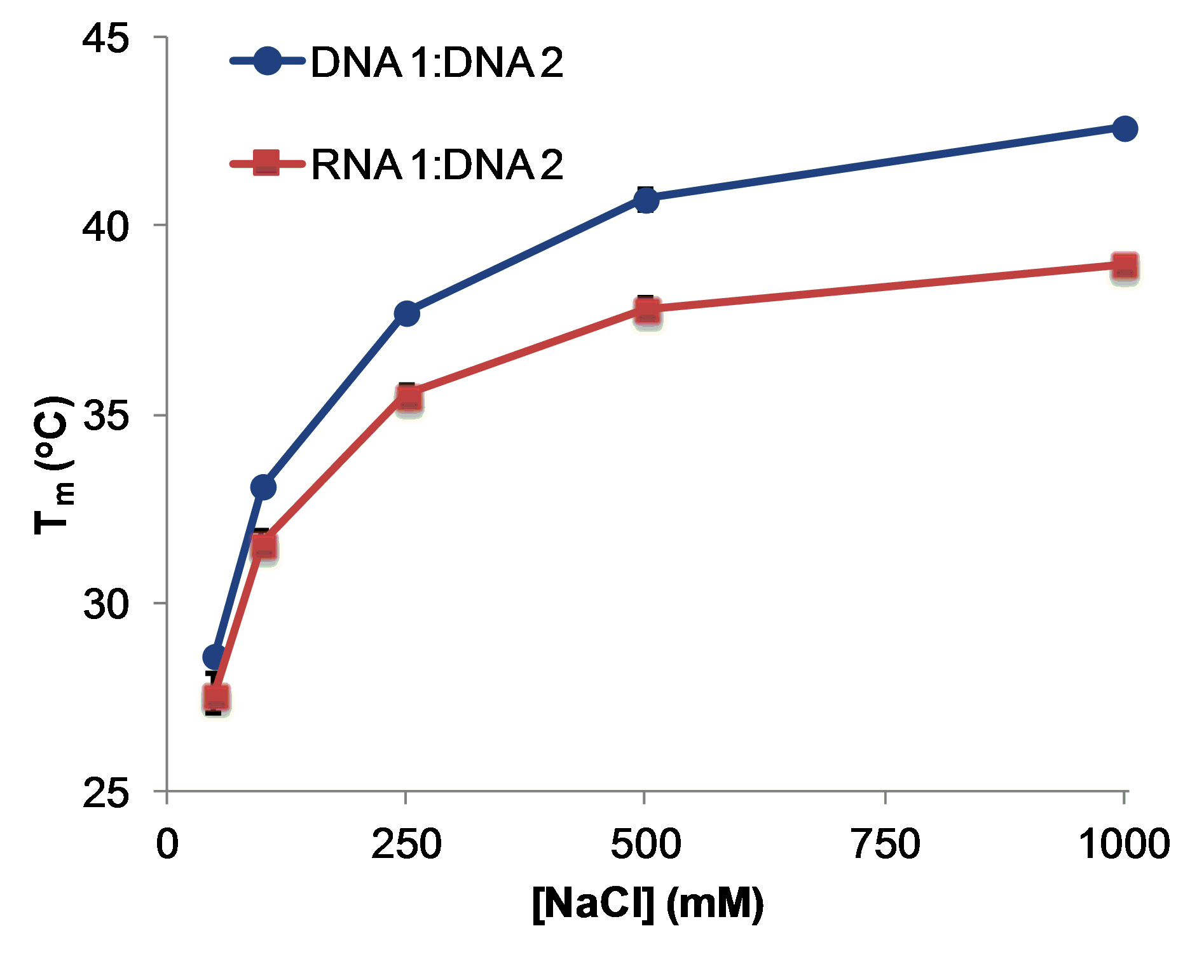

Supplement: Figure S1 — Tm vs [NaCl] for DNA 1:DNA 2 and RNA 1:DNA 2 duplexes. Conditions: 3 µM DNA, 3 µM RNA, 10 mM phosphate buffer with added NaCl, pH 7.2. Error bars represent standard deviation of three independent trials. (TIF) [file pone.0058670.s001.tif]

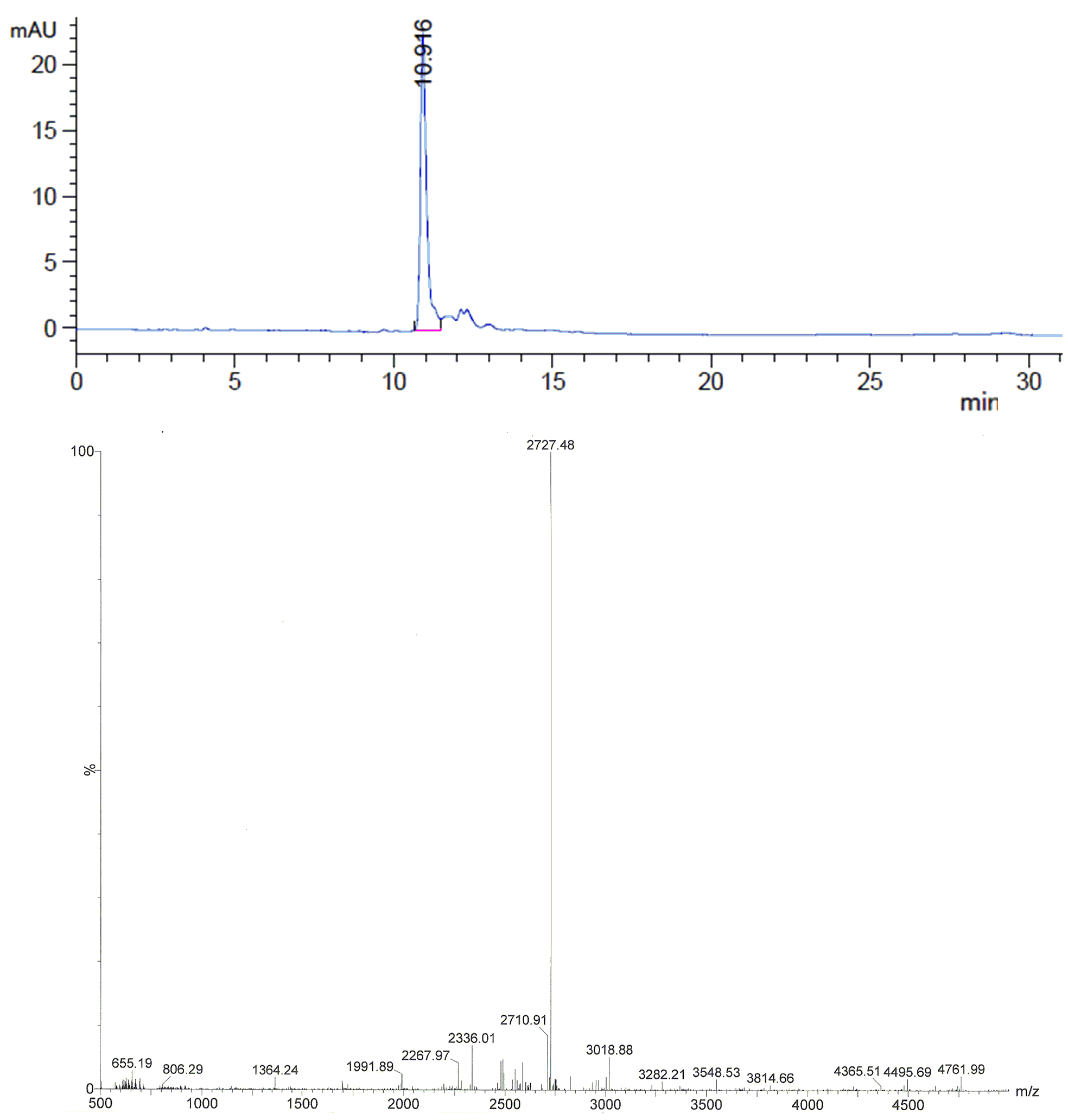

Supplement: Figure S2 — HPLC and MALDI-TOF MS of PNA nf (H-GTAGATCACT-NH2). m/z 2727.48 (calcd [M]+ 2727.04). (TIF) [file pone.0058670.s002.tif]

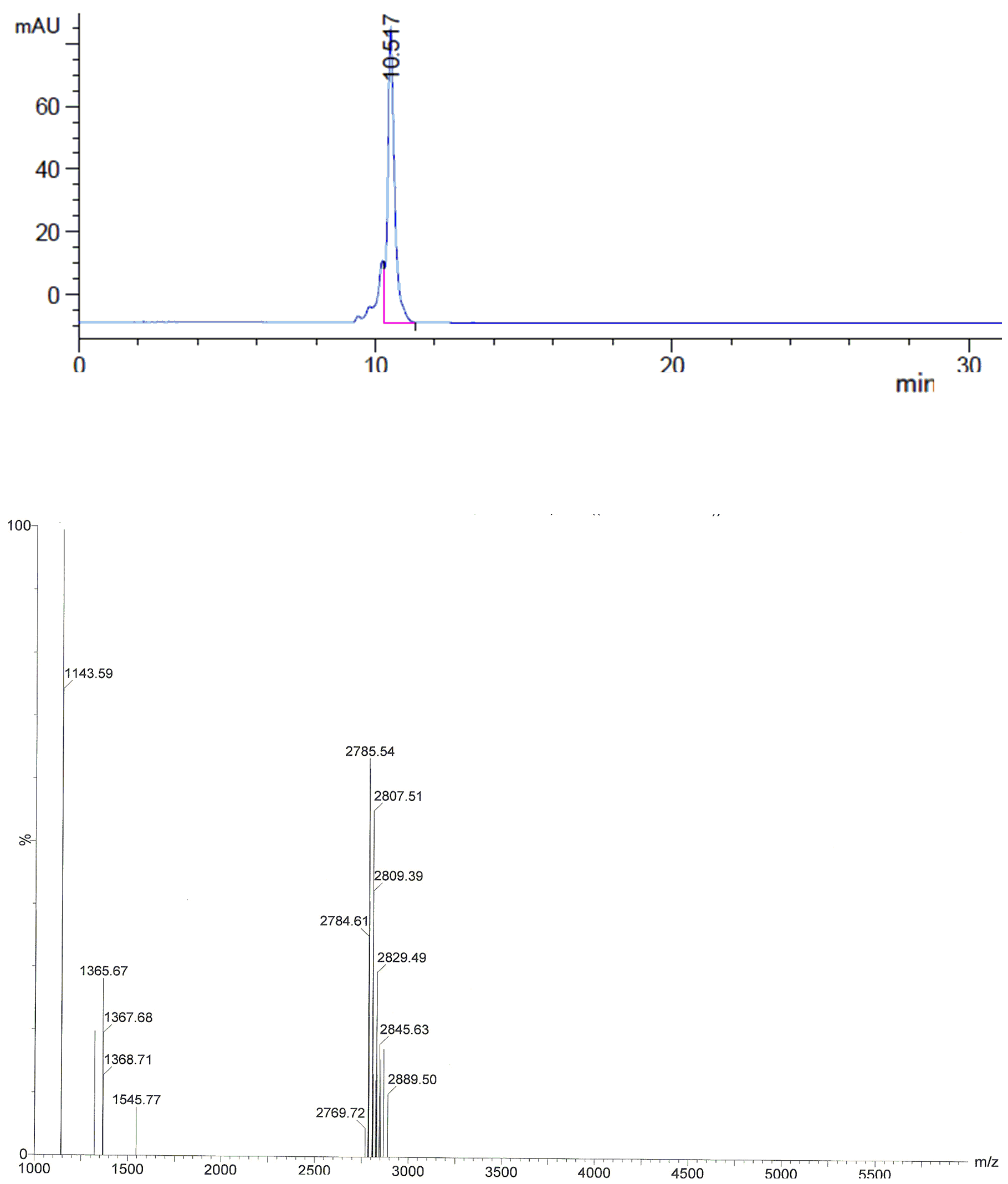

Supplement: Figure S3 — HPLC and MALDI-TOF MS of PNA 1neg (H-GTAGATDCACT-NH2). m/z 2785.54 (calcd [M]+ 2785.04). (TIF) [file pone.0058670.s003.tif]

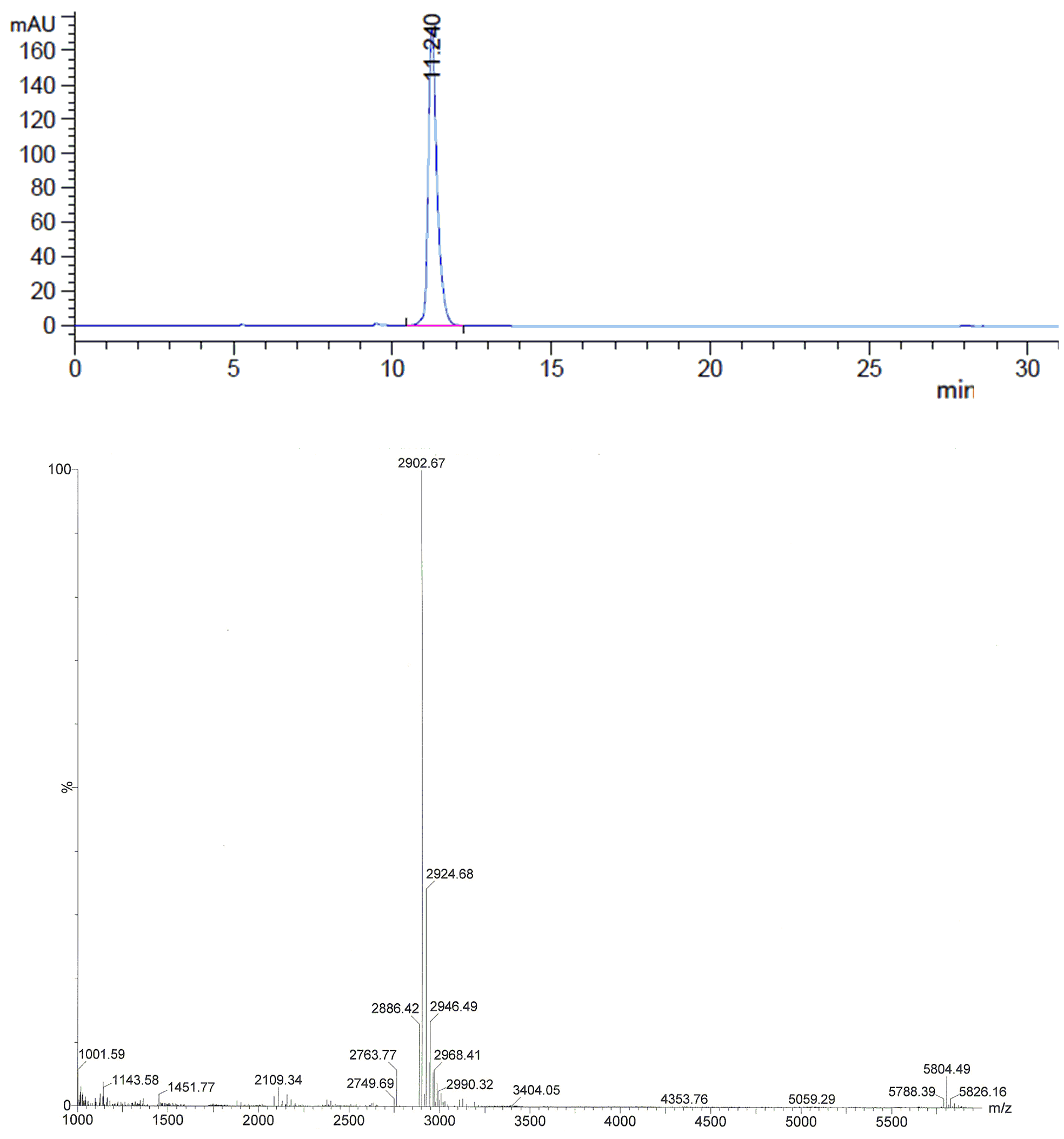

Supplement: Figure S4 — HPLC and MALDI-TOF MS of PNA 3neg (H-GTDAGATDCACTD-NH2). m/z 2902.67 (calcd [M+H]+ 2902.14); 2924.68 (calcd [M+Na]+ 2924.12). (TIF) [file pone.0058670.s004.tif]

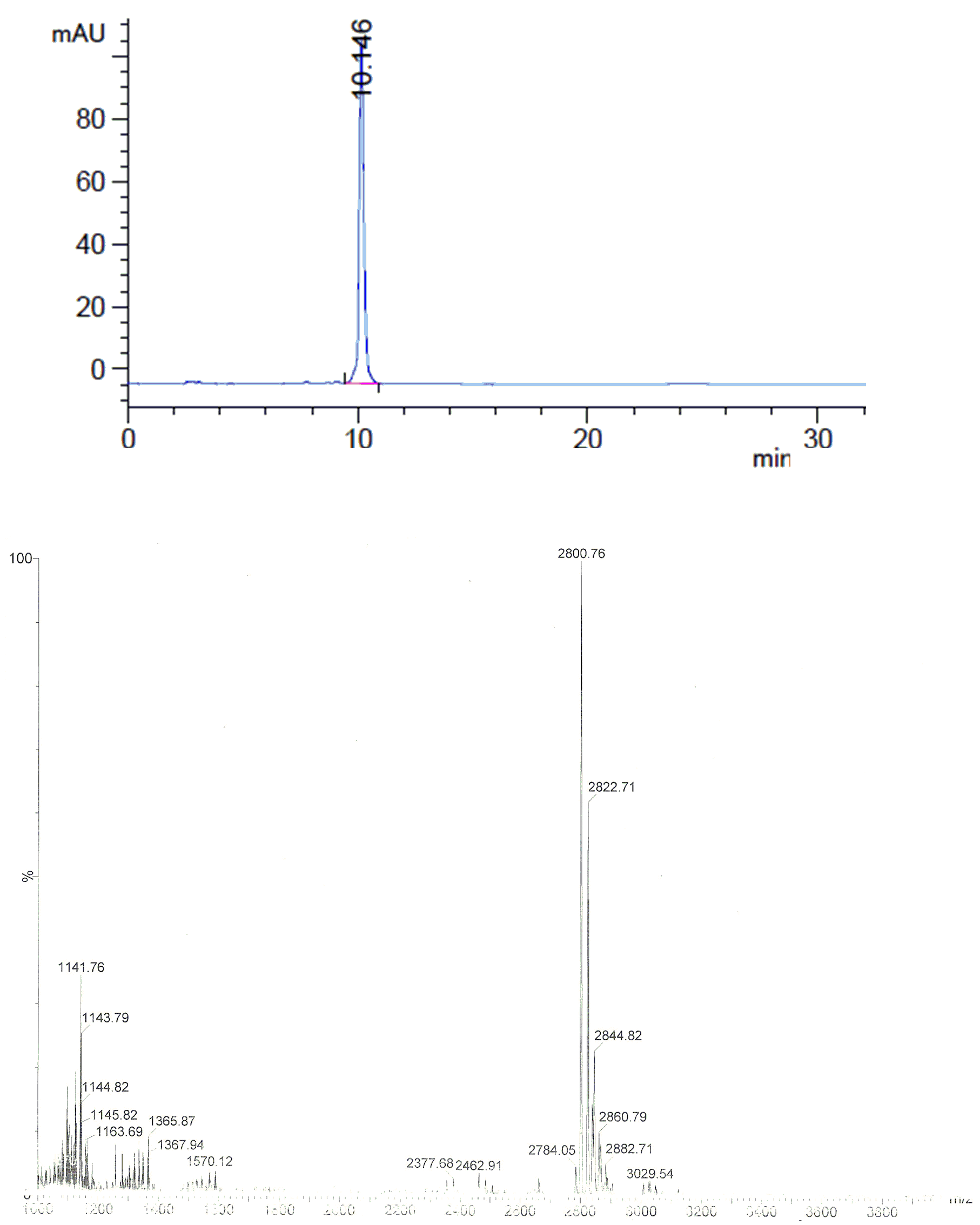

Supplement: Figure S5 — HPLC and MALDI-TOF MS of PNA 1pos (H-GTAGATKCACT-NH2). m/z 2800.76 (calcd [M+H]+ 2799.12); 2822.71 (calcd [M+Na]+ 2821.1). (TIF) [file pone.0058670.s005.tif]

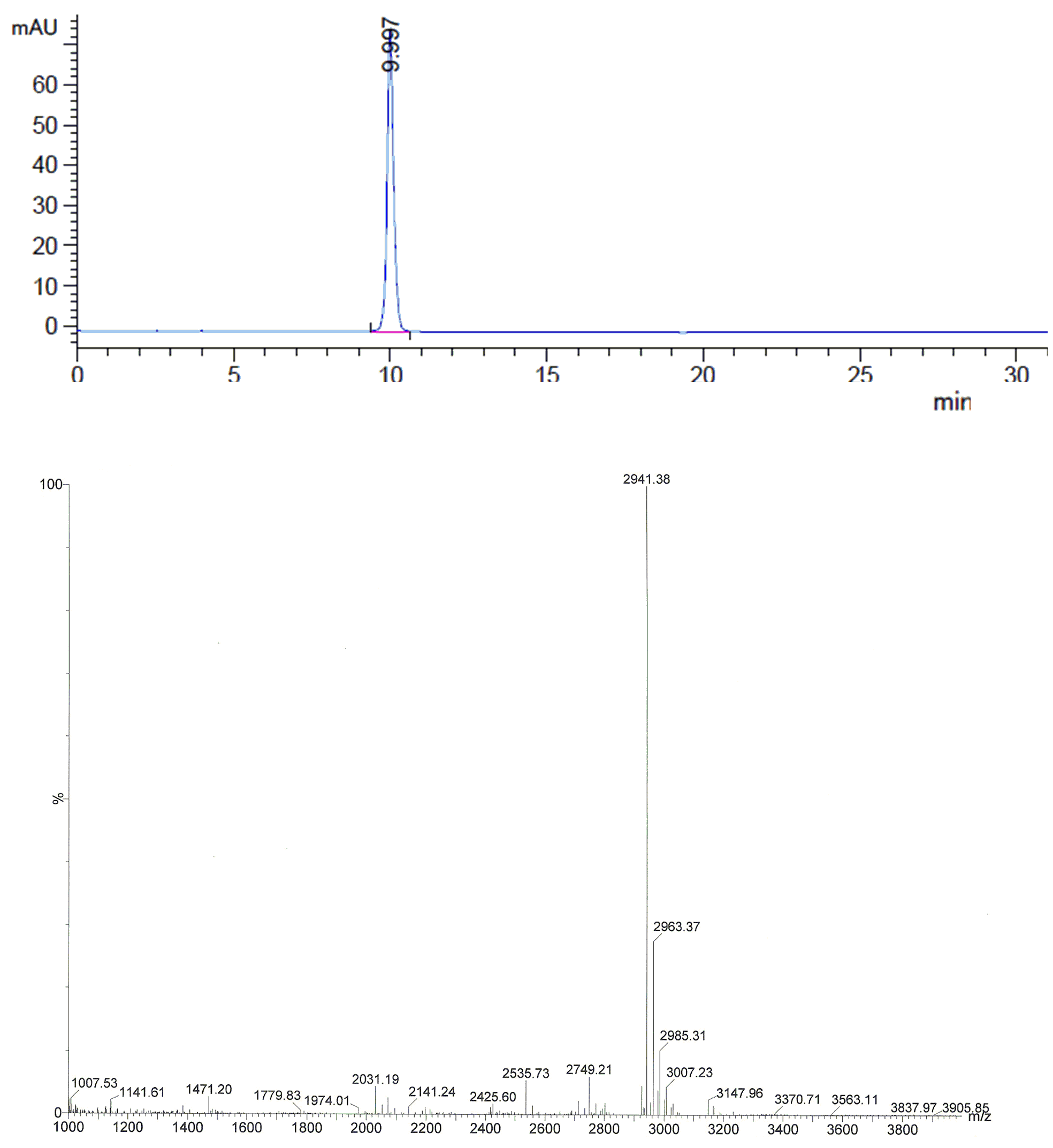

Supplement: Figure S6 — HPLC and MALDI-TOF MS of PNA 3pos (H-GTKAGATKCACTK-NH2). m/z 2941.38 (calcd [M+H]+ 2941.26); 2963.37 (calcd [M+Na]+ 2963.24). (TIF) [file pone.0058670.s006.tif]

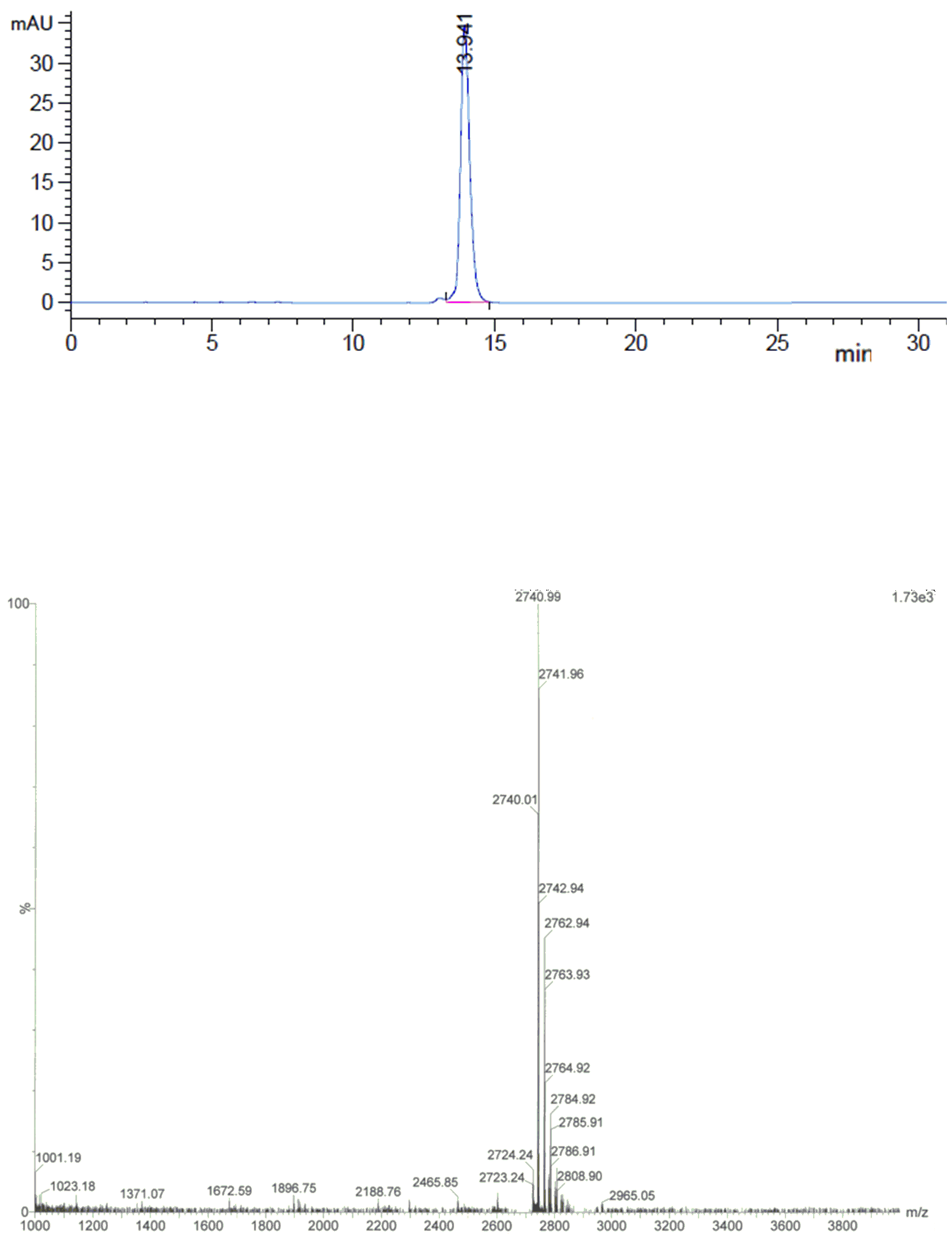

Supplement: Figure S7 — HPLC and MALDI-TOF MS of PNA 1Me (H-GTAGATACACT-NH2). m/z 2740.99 (calcd [M]+ 2741.05); m/z 2741.96 (calcd [M+H]+ 2942.06); 2763.93 (calcd [M+Na]+ 2764.04). (TIF) [file pone.0058670.s007.tif]
