# Supplementary material for: Evaluating the Effect of Ionic Strength on Duplex Stability for PNA Having Negatively or Positively Charged Side Chains
Source: PLoS One. 2013 Mar 6;8(3):e58670. doi: 10.1371/journal.pone.0058670 (PMC3590165; doi:10.1371/journal.pone.0058670)
Supplement: File S1 — General techniques and synthesis of PNA monomers. (DOC) [file pone.0058670.s008.doc]

**General techniques**. Glassware for all reactions was oven dried or flame dried and cooled prior to use. All reactions were run under an atmosphere of nitrogen or argon unless otherwise stated. Tetrahydrofuran (THF) was distilled from sodium and benzophenone. Dichloromethane (CH_2_Cl_2_) and dimethylformamide (DMF) were passed through a solvent purification system (J. C. Meyer). Unless otherwise noted, all starting materials were obtained from commercial suppliers and were used without further purification. Thin layer chromatography was performed on Silica gel 60 F_254_ plates eluting with the solvent indicated, visualized by a 254/365 nm UV lamp, or stained with a solution of ninhydrin or potassium permanganate. Column chromatography was performed on Merck silica gel Kieselgel 60 (230-400 mesh, 40–63 μm particle size). Yields were calculated for material judged homogenous by thin layer chromatography and NMR. Compounds were named using CS ChemBioDraw Ultra 12.0. NMR spectra were acquired on a Varian Unity-300 or VXR 500 spectrometer. Chemical shifts for ^1^H NMR spectra are reported in parts per million relative to the signal of residual CHCl_3_ at 7.27 ppm or the center line of the residual acetone pentet at 2.05 ppm. Chemicals shifts for ^13^C NMR spectra are reported in parts per million relative to the center line of the CDCl_3_ triplet at 77.23 ppm or the center line of the acetone septet at 29.9 ppm. The abbreviations s, d, dd, t, q, m, br, and rot stand for singlet, doublet, doublet of doublets, triplet, multiplet, broad, and rotamer respectively. IR spectra were obtained from Nicolet 380 FT-IR spectrometer. Optical rotations were obtained at ambient temperature on a Perkin Elmer Model 343 polarimeter (Na D line) using a microcell with a 1 decimeter path length, and the reported concentrations (c) are in g/100 ml. Mass spectra were recorded at the Mass Spectrometry facility in the Department of Chemistry of the University of Utah. DNA/RNA was purchased from the University of Utah DNA/Peptide Synthesis Core Facility. Milli-Q water was obtained from Millipore Simplicity UV water purification system.

**Synthesis of the monomers.**

**(S)-tert-butyl3-((((9H-fluoren-9-yl)methoxy)carbonyl)amino)-4-((2-(benzyloxy)-2-**

**oxoethyl)amino)butanoate 4.** To a solution of aldehyde **3**(48) (1.611 mmol) in CH_2_Cl_2_ (20 ml), glycine benzyl ester 4-toluenesulfonate (0.652 g, 1.933 mmol) and Et_3_N (0.67 ml, 4.83 mmol) were added at 0 ºC. After stirring for 30 min, sodium triacetoxyborohydride (0.444 g, 2.094 mmol) was added and the reaction mixture was stirred at RT for 16 h. The reaction was quenched with sat. NaHCO_3_ (12 ml) and sat. Na_2_CO_3_ (4 ml). After stirring for 30 min, the mixture was extracted with CH_2_Cl_2_ (3 x 30 ml). The combined organic extracts were washed with brine, dried over Na_2_SO_4_, and concentrated. The crude material was purified by silica flash column chromatography (1:1 EtOAC:Hexane) to afford 0.526 g (60%, two steps from **2**) of the secondary amine **4** as a pale yellow oil (R_f_ = 0.4 in 1:1 EtOAC:Hexane); [α]_D_^25^ = +0.4^o^ (c 0.9, CH_2_Cl_2_); ^1^H NMR (300 MHz, CDCl_3_) δ 7.67 (d, *J* = 7.4 Hz, 2 H), 7.54-7.5 (m, 2 H), 7.33-7.17 (m, 9 H), 5.54 (br d, *J* = 8.4 Hz, 1 H), 5.08 (s, 2 H), 4.31 (d, *J* = 6.7 Hz, 2 H), 4.14 (t, *J* = 6.7 Hz, 1 H), 3.97 (br s, 1 H), 3.38 (d, *J* = 3.7 Hz, 2 H ), 2.78-2.62 (m, 2 H), 2.51-2.38 (m, 2 H), 1.88 (br s, 1 H), 1.37 (s, 9 H); ^13^C NMR (125 MHz, CDCl_3_) 172.2, 170.9, 156.1, 143.8, 141.2, 135.5, 128.5, 128.32, 128.27, 127.6, 127, 125, 119.9, 81, 66.6, 64, 52, 50.5, 50.1, 47.2, 37.2, 28; IR (neat): 3330, 2975, 1724, 1521, 1450, 1251, 1158 cm^-1^; HRMS (ESI) *m/z* for C_32_H_36_N_2_O_6_: 567.2477 (calcd [M+Na]^+^ 567.2471).

**(S)-tert-butyl3-((((9H-fluoren-9-yl)methoxy)carbonyl)amino)-4-(N-(2-(benzyloxy)-**

**2-oxoethyl)-2-(5-methyl-2,4-dioxo-3,4-dihydropyrimidin-1(2H)-yl)acetamido)butano**

**ate 5.** To a solution of thymine-1-acetic acid (0.059 g, 0.32 mmol), HATU (0.122 g, 0.320 mmol) and 4 Å molecular sieves in DMF (0.9 ml) at 0 °C, DIPEA (0.060 ml, 0.32 mmol) was added. After stirring at 0 °C for 15 min, a solution of secondary amine **4** (0.146 g, 0.267 mmol) in CH_2_Cl_2_ (1.5 ml) was added and stirred at 0 °C for 10 min. After stirring at RT for 16 h, the reaction was concentrated and washed with H_2_O. The aqueous layer was extracted with EtOAc (3x). The combined organic extracts were washed with sat. NH_4_Cl, sat. NaHCO_3_, and brine, dried over Na_2_SO_4_, and concentrated. The crude mixture was purified by silica flash column chromatography (a gradient of EtOAC:Hexane from 3:7 to 8:2) to afford 0.097 g (51%) of amide **5** as a pale yellow oil (R_f_ = 0.5 in 8:2 EtOAC:Hexane) and 0.037 g (20%) starting secondary amine **4**; [α]_D_^25^ = +5.5^o^ (c 0.2, CH_2_Cl_2_); ^1^H NMR (500 MHz, CDCl_3_) δ 9.7 (s, 0.6 H, rot 1), 9.66 (s, 0.4 H, rot 2), 7.62 (d, *J* = 7.7 Hz, 2 H), 7.49-7.46 (m, 2 H), 7.31-7.24 (m, 5 H), 7.23-7.15 (m, 4 H), 6.83 (s, 0.6 H, rot 1), 6.73 (s, 0.4 H, rot 2), 6.27 (d, *J* = 8.4 Hz, 0.5 H, rot 1), 5.81 (d, *J* = 8.4 Hz, 0.4 H, rot 2), 5.1 (s, 0.8 H, rot 1), 5.0 (s, 1.2 H, rot 2), 4.56 (s, 1 H, rot 1), 4.34-4.22 (m, 3 H), 4.2-4.0 (m, 4 H), 3.6-3.4 (m, 2 H), 2.56-2.49 (m, 1.2 H, rot 1), 2.44-2.34 (m, 0.8 H, rot 2), 1.73 (s, 1.7 H, rot 1), 1.72 (s, 1.3 H, rot 2), 1.34 (s, 5 H, rot 1), 1.32(s, 4 H, rot 2); ^13^C NMR_major rotamer_ (125 MHz, CDCl_3_) 170.7, 169.1, 168.5, 168, 156.4, 151.5, 144, 143.8, 141.4, 135.2, 128.9, 128.7, 128.3, 127.8, 127.2, 125.2, 120.1, 110.7, 82, 67.9, 67.3, 51, 49.2, 47.9, 47.4, 47.2, 37.4, 28.1, 12.4; IR (neat): 3335, 2924, 1720, 1522, 1450, 1248, 1155 cm^-1^; HRMS (ESI) *m/z* for C_39_H_42_N_4_O_9_: 733.2841 (calcd [M+Na]^+^ 733.2849).

**(S)-2-(N-(2-((((9H-fluoren-9-yl)methoxy)carbonyl)amino)-4-(tert-butoxy)-4-oxobutyl**

**)-2-(5-methyl-2,4-dioxo-3,4-dihydropyrimidin-1(2H)-yl)acetamido)acetic acid 6.** To a solution of amide **5** (0.098 g, 0.14 mmol) in MeOH (5 ml) was added Pd/C (0.010 g) under N_2_. The flask was evacuated, then flushed with H_2_ gas. The reaction was placed under H_2_ (balloon pressure). After stirring for 16 h, the reaction mixture was filtered through celite and evaporated under reduced pressure to give 0.068 g (80%) of the modified peptide nucleic acid monomer **6** as a white solid (R_f_ = 0.1 in 9:1 CH_2_Cl_2_:MeOH); mp = 88-90^o^C; [α]_D_^25^ = +2.4^o^ (c 0.5, CH_3_OH); ^1^H NMR (500 MHz, acetone-d6) δ 10.67 (br s, 1 H), 7.82 (d, *J* = 6.8 Hz, 2 H), 7.66 (d, *J* = 4.9 Hz, 2 H), 7.38-7.23 (m, 4 H), 6.95 (br s, 0.5 H, rot 1), 6.7 (br s, 0.5 H, rot 2), 4.92-4.76 (br m, 1 H), 4.59 (br s, 1.6 H, rot 1), 4.43 (br s, 0.4 H, rot 2), 4.38-4.32 (partially obscured br m, 1.4 H, rot 1,2), 4.28 (partially obscured br s, 1.6 H, rot 1), 4.24-4.1 (partially obscured br m, 2 H), 3.91 (dd, *J* = 6.8 Hz, 0.5 H, rot 1), 3.64-3.54 (partially obscured m, 1 H, rot 1,2), 3.39 (dd, *J* = 6.8 Hz, 0.5 H, rot 2), 2.6-2.48 (m, 2 H), 1.74 (br s, 3 H), 1.42 (s, 4 H, rot 1), 1.39(s, 5 H, rot 2); ^13^C NMR_major rotamer_ (125 MHz, acetone-d6) 171, 169.4, 168.8, 165.5, 157.1, 152.5, 144.8, 143.1, 141.9, 128.5, 127.8, 126.1, 120.8, 110.3, 81.2, 67.4, 52 (br), 49.2 (br), 48.4, 47.9, 47.8, 43, 28.2, 12.3; IR (neat): 2978, 1662, 1477, 1230, 1155 cm^-1^; HRMS (ESI) *m/z* for C_32_H_36_N_4_O_9_: 643.2396 (calcd [M+Na]^+^ 643.2380).
